# Healthy city: global systematic scoping review of city initiatives to improve health with policy recommendations

**DOI:** 10.1186/s12889-023-15908-0

**Published:** 2023-07-01

**Authors:** Shaun Danielli, Hutan Ashrafian, Ara Darzi

**Affiliations:** 1 Kings Health Partners, Guys Hospital, London, SE1 9RT UK; 2 Imperial College London, South Kensington Campus, London, SW7 2NA UK

**Keywords:** City, Urban, Health, Transformation, Systematic

## Abstract

**Background:**

Global health will increasingly be determined by cities. Currently over half of the world’s population, over 4 billion people, live in cities. This systematic scoping review has been conducted to understand what cities are doing to improve health and healthcare for their populations.

**Methods:**

We conducted a systematic search to identify literature on city-wide initiatives to improve health. The study was conducted in accordance with PRISMA and the protocol was registered with PROSPERO (CRD42020166210).

**Results:**

The search identified 42,137 original citations, yielding 1,614 papers across 227 cities meeting the inclusion criteria. The results show that the majority of initiatives were targeted at non-communicable diseases. City health departments are making an increasing contribution; however the role of mayors appears to be limited.

**Conclusion:**

The collective body of evidence identified in this review, built up over the last 130 years, has hitherto been poorly documented and characterised. Cities are a meta-system with population health dictated by multiple interactions and multidirectional feedback loops. Improving health in cities requires multiple actions, by multiple actors, at every level. The authors use the term ‘The Vital 5’. They are the five most important health risk factors; tobacco use; harmful alcohol use; physical-inactivity, unhealthy diet and planetary health. These ‘Vital 5’ are most concentrated in deprived areas and show the greatest increase in low and middle income countries. Every city should develop a comprehensive strategy and action plan to address these ‘Vital 5’.

**Supplementary Information:**

The online version contains supplementary material available at 10.1186/s12889-023-15908-0.

## Background

Global heath will increasingly be determined by cities. Currently over half (55 per cent) of the world’s population lives in cities and by 2050, 68 percent of the world’s population is projected to be urban [1]. This growth has occurred and will continue to occur over a relatively short period. The world’s cities are growing in both size and number in all regions [2] but there is particularly rapid growth in the number and size of cities across Asia, Africa and South America [3]. For example, between 1950 and 2005, the percentage of the population of China living in cities rose from 13 to 40% and it is predicted to rise to 60.3 percent by 2030 [2]. To put the scale of the urbanisation challenge and opportunity in context, since 2003, China poured more cement *every two *years than *the *US *did in the *entire 20th century [4]. Concrete manufacture accounts for at least 8% of global carbon emissions [5]. This will have a significant impact on environmental health which has both direct and indirect impact on population health.

Whilst cities contain some of the best health and healthcare, they also contain a disproportionate share of the worst [6]. There also remains significant variation in health and high levels of inequality [7]. Many urban health and environmental challenges are a consequence of how we organise and live in cities [8] which have resulted in cities that are making people sicker, fatter and more socially isolated [9–11]. Globally cities have been hardest hit by Covid-19 [12].

However, there are also reasons for optimism. Cities are the world's engines of economic growth, innovation, and social change [13]. City living is better for the environment [3] and city dwellers have had better health than their rural counterparts since at least the early to mid-20th century, in high income as well as low and middle income countries [6, 14] Climate change is the number one threat to population health [15–18] and it is cities that are leading the way in becoming carbon neutral [19]. Cities have been shown to succeed—where national governments have failed—in making significant improvements in areas affecting population health [20–26].

It is widely accepted we need to improve equitable health and care in cities. There is good financial imperative to do so; good health is not only a consequence of, but also a condition for sustained and sustainable economic development [27]. In this way, the health of a city creates a virtuous circle of improved health and improved economic prosperity. Not just for the city but for its nation [14]. There is a compelling case to improve health in city populations.

The aim of this global systematic scoping review is to provide an overview of the existing evidence of city initiatives to improve health. In addition, a summary of each of the 1614 papers which describe an initiative to improve health or healthcare is provided (Additional file 1) in a format that can be thematically searched. This will be a useful resource for academics and policy makers interested in improving urban health. The outputs of this systematic scoping review should be used to inform urban health policy for the next decade.

## Methods

### Aim and overview

A systematic search was conducted to identify literature on city-wide initiatives to improve health. This study was completed in accordance with the PRISMA extension for scoping reviews [28] The protocol was registered at PROSPERO (number CRD42020166210).

### Search strategy and selection criteria

**Fig. 1:**
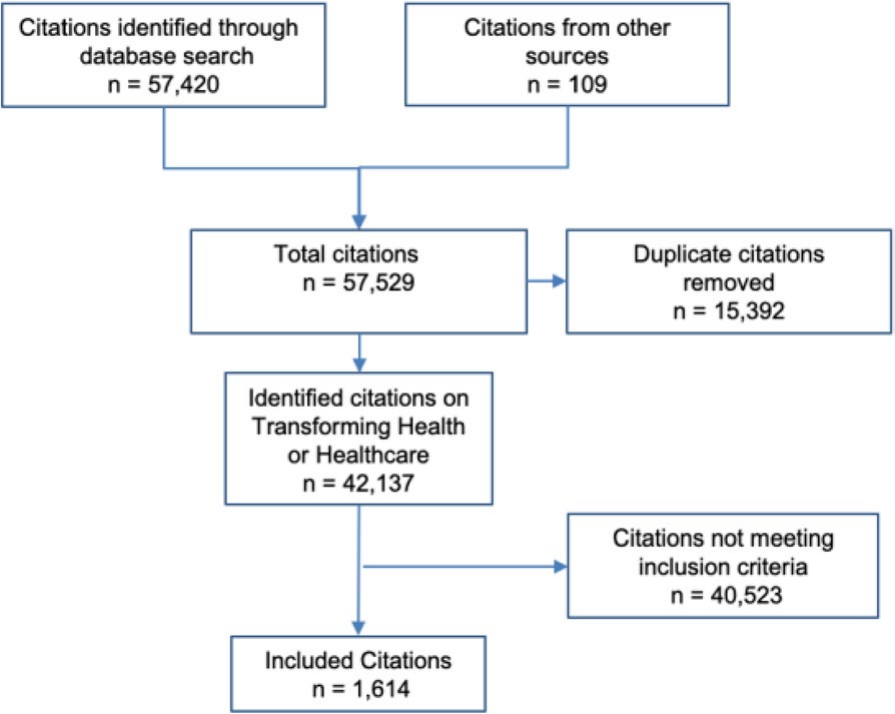
– PRISMA [29] study attrition flow chart

Ten databases were searched; Embase, Ovid Medline, Cochrane Database (CENTRAL), Scopus, Campbell Library, CINALH, Health Business Elite, Health Management Information Consortium (HMIC), PyschINFO and Prospero. Specific and pre-determined search terms were developed, tested and finalised for each database. These are available in Additional file 1. The inclusion criteria was: city-wide initiatives that aimed to improve health or healthcare. The definition of improvement, for the purposes of this research, was purposefully broad and included any initiative whose aim was to improve health outcomes or healthcare services. Citations that did not describe an improvement initiative or did describe an improvement initiative but only of a single organisation and not city-wide were excluded. City-wide improvement initiative of family planning services and malaria and dengue fever were excluded. Citations not in English language were also excluded. All databases were searched on the 10 – 12 February 2016, for entries from database inception to February 2016 (tranche 1). The search was updated on 16 March 2019, for entries from February 2016 to March 2019 (tranche 2). Extensive hand searching was also completed. The database results were uploaded to Endnote (version 9) (Fig. 1).

### Publication selection

All types of study design were eligible for inclusion, including grey literature. Studies were not selected or screened based on methodological quality. The key factor was that the study needed to describe a city-wide initiative to improve health or healthcare. All initiatives were included irrespective of how ambitious they were. The primary outcome indicators that were sought and extracted were: improved health or reduced health inequalities; improved healthcare (safety, effectiveness, patient experience); failed initiative or negative or unintended consequences. The first tranche (31,251 original citations) were assessed independently by the lead author (SD) and HLP^1^ against the inclusion criteria. The level of inter-reviewer agreement on the articles was on average 96% (range 91%—98%). Where there was a disagreement, the two reviewers discussed and agreed on a final outcome. The second tranche was assessed by SD. Where it was uncertain whether the initiatives were city-wide, they were included.

### Data analysis

Predetermined data fields were agreed prior to data extraction. The data fields were; date of publication; type of publication; initiative; how the initiative was implemented; city; city population; country; continent; quantitative outcome; author’s qualitative conclusion. A template (excel) was developed for data extraction and analysis. Where any of this data was not presented, it was coded as ‘not stated’. The only exception to this was with city population. Where this was not presented, the information was sought through world statistics organisation [30]. The latest available population data was used (irrespective of when the initiative took place). The lead author (SD) extracted the data and systematically coded and analysed the data. Studies that did not name a specific city were still included as long as that they indicated they were from a city. Inductive thematic analysis, in line with the guidelines of Braun and Clarke, [31] was carried out as it offered optimum flexibility and breadth. The included studies were not critically appraised for quality. However, the authors have highlighted throughout the text, major methodological issues, source of study heterogeneity and necessary contextual information for interpreting the results. This data was used by the authors to debate and agree the key findings and recommended policy actions.

### Patient and public involvement

This research was done without patient or public involvement.

## Results

The database searches yielded a total of 42,028 original citations after duplicates were removed through Endnote standard duplicate check. These electronic searches were augmented with an additional 109 citations found through expert recommendations or internet searches, giving a total of 42,137 original citations. These citations were assessed and resulted in 1614 citations meeting the inclusion criteria.

The full text of the 1614 citations was screened and all included in the inductive thematic coding. The overwhelming majority (90%, *n* = 1455) of papers were journal articles. The publication date ranged from 1892 to 2019.

### Subject areas of health improvement

**Fig. 2:**
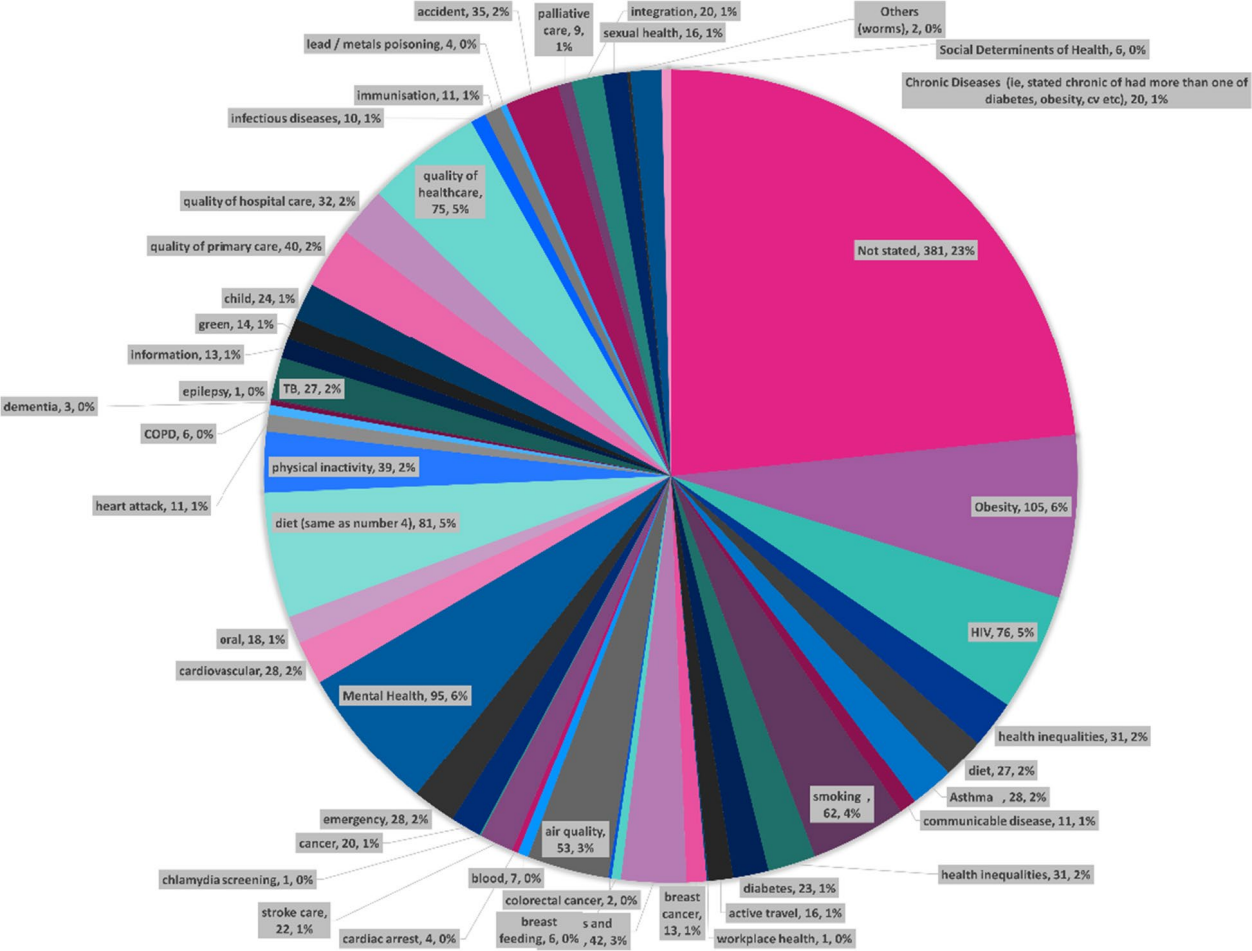
Subject area cities were seeking to address

A total of 47 themes emerged as subject areas that cities were seeking to address. Just over two thirds (69% *n* = 1113) of the subject areas were related to improving health, with the remaining 31% (*n* = 501) related to improving health services. Across improving health and improving healthcare, 92% (*n* = 1483) were addressing non-communicable diseases (Fig. 2).

**Fig. 3:**
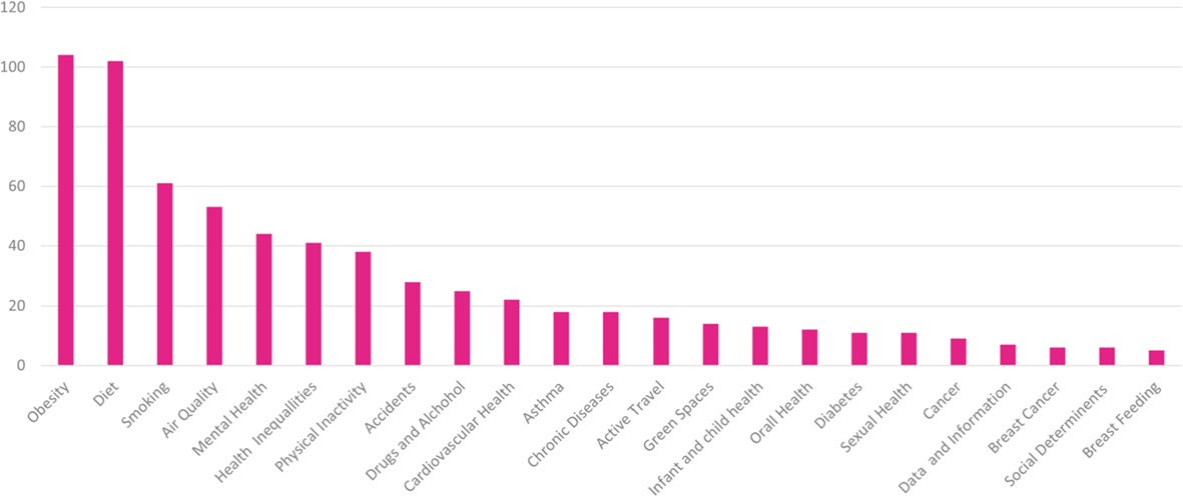
Non-communicable health subject areas that cities were seeking to address

Of those related to improving health, 1043 (94%) were focused on non-communicable diseases and 70 (6%) were focused on communicable diseases. In relation to non-communicable diseases, there were 39 different subject areas addressed. A significant proportion (*n* = 354 equivalent to 33%) did not state a specific area of health they were seeking to address. Where a specific area was stated, the highest was obesity (*n* = 104), followed by diet (*n* = 102), smoking (*n* = 61) and then air quality (*n* = 53). The non-communicable health subject areas are presented in Fig. 3 below. The communicable diseases [32] included; HIV (*n* = 44), TB (*n* = 12), Sexual Transmitted Disease (*n* = 1), Influenza, Small Pox, Typhoid and others (*n* = 10).

This graph shows subject areas included in five or more citations. Those coded as general health or not stated have been removed.

**Fig. 4:**
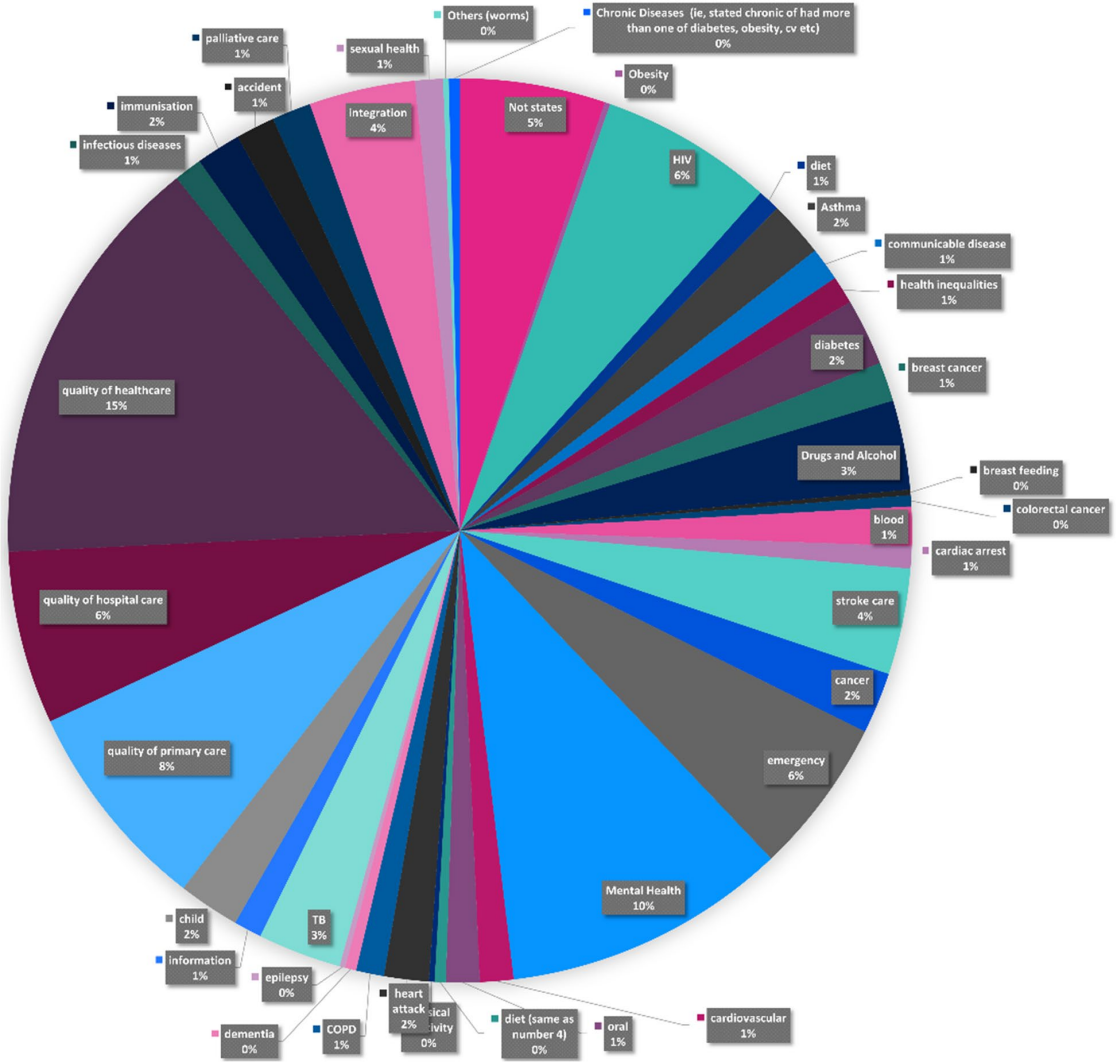
Subject areas that were being addressed through improving healthcare

Those relating to improving health services (*n* = 501) were split across forty thematic areas as shown in Fig. 4 below.

### Type of initiative

**Fig. 5:**
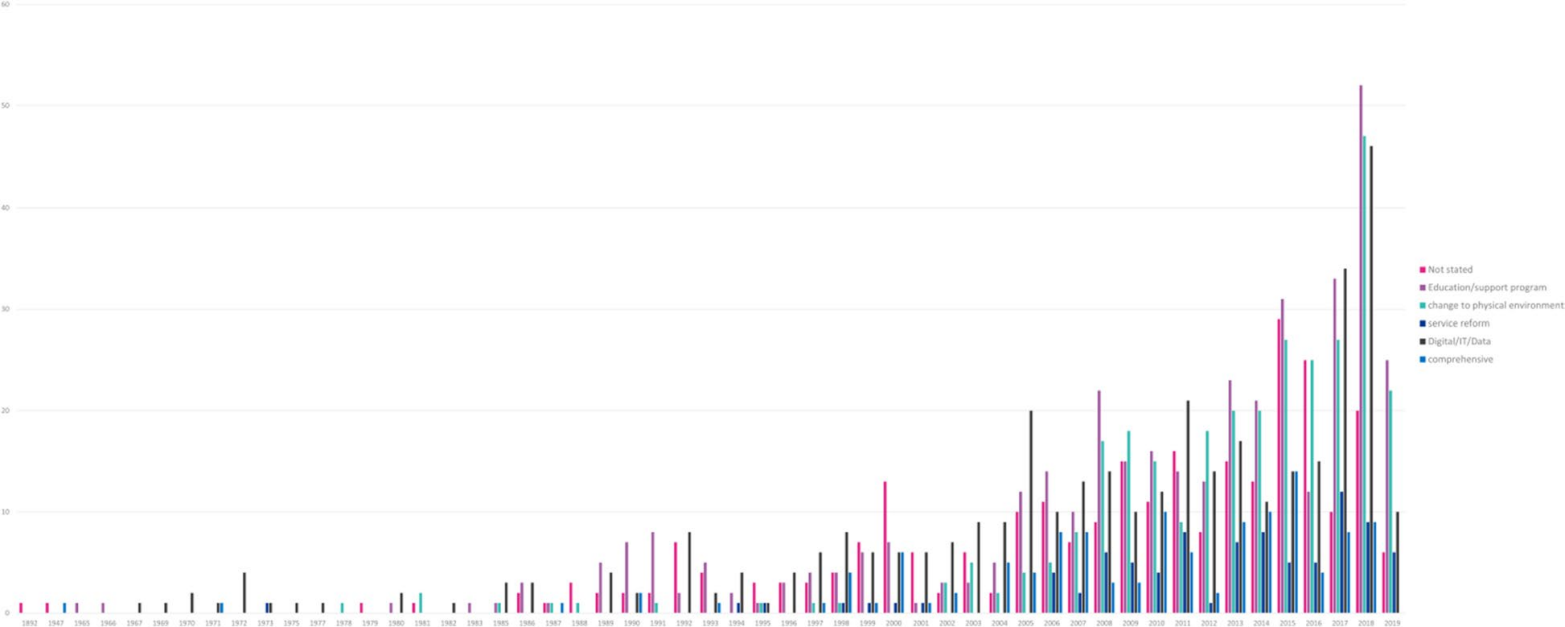
Type of initiative per year

There were 6 categories of initiative identified (Table 1). Education was the most frequently applied type of initiative with 26%, followed closely by service reform (23%) and change to physical environment (21%). A significant proportion (17%) either did not state the type of initiative or it was unclear. Less than a tenth (8%) described a comprehensive initiative and the smallest volume of initiatives were related to digital, data or IT (6%) (Fig. 5).

**Table 1:** Type of initiative

**Category**	**Definition of category**	# of papers	%
Education / support program	A programme to education or support the individual(s), either patient / population or workforce. For example, the education initiative to improve healthy lifestyles with a focus on school children [33]	414	26%
Service reform	A change or addition to a health service, ie improved patient flow redesign or additional outpatient clinic. For example, the description of Shanghai’s service reforms [34]	366	23%
Change to physical environment	A change to the physical environment to change the world in which the individual interacts. For example, Los Angeles banning fast-food restaurants [35]	336	21%
Comprehensive	The initiative was described as, and believed to be, a comprehensive initiative (ie more than one single initiative) and / or it contained more than one of these 6 categories. For example, the urban health strategy in Detroit [36]	124	8%
Digital / IT / Data	A digital or data initiative, or the use of IT more generally, to improve health or healthcare	92	6%
Not stated / unclear	There was not an initiative or if there was it was not stated in the title or abstract. For example, Taipei’s multi-channel digital risk communications to prevent the spread of a communicable disease [37]	282	17%

#### How the initiative was instigated

**Fig. 6:**
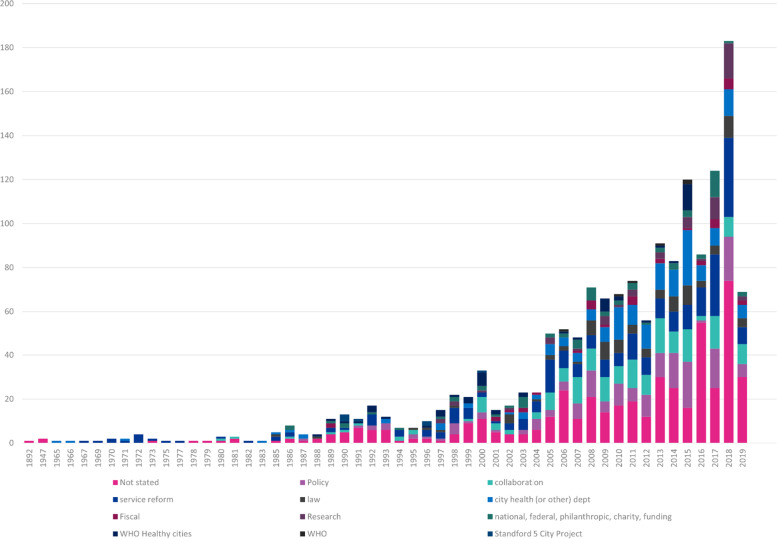
How initiatives were instigated each year

There were 12 categories which indicated how the initiative was instigated (Table 2). Eight were predetermined ahead of data extraction, four (national or philanthropic funded, research, WHO, Stanford 5 city project) were identified during extraction. Over a third (34%) did not state how the initiative was instigated. Policy (12%), collaboration (12%), service reform (11%) and law (11%) were the next highest methods of instigating an initiative. It is worth noting the contribution of City Health Departments (6%), WHO Healthy Cities (5%) and national or philanthropic (5%). Only 44 (3%) referenced a Mayor as having a significant role (Figure 6) (Table 2).

**Table 2:** Number of papers in each category

Categories of ‘how’ initiative was instigated	# of papers	%
Policy	178	11%
Collaboration	181	11%
Service Reform	247	15%
Law	91	6%
City Health (or other) Department	163	10%
WHO Healthy Cities	51	3%
National or Philanthropic	68	4%
Fiscal / Financial levers	38	2%
Research	55	3%
WHO (but not Healthy Cities)	6	0%
Stanford 5 City Project	7	0%
Not stated	529	33%

#### Location

The city in which an initiative was carried out was recorded and coded, as was the country and continent of the city. In addition, the population of the city was recorded if it was stated and if it was not recorded it was sought. The 1614 citations represent initiatives from 227 cities. The majority of cities were just in one paper (*n* = 179 cites). New York City accounted for nearly one fifth (17% 279 of 1614) of the total papers, followed by Baltimore (51) and London (48). Collectively, these 3 cities (less than 1% of total number of cities) account for nearly a quarter (23%) of all the citations.

**Fig. 7:**
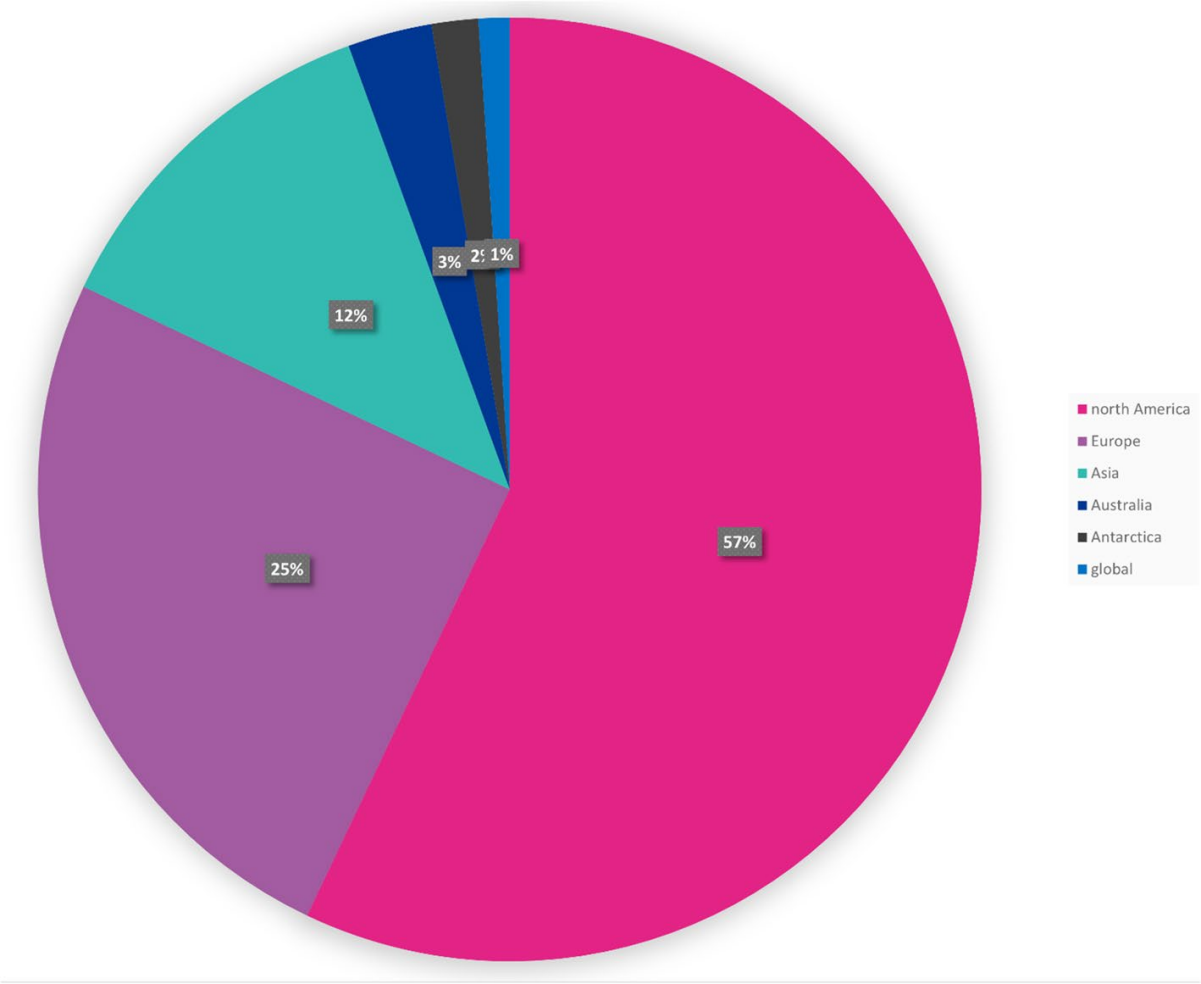
Citations per continent

The 227 cities come from 80 countries across five continents. The USA accounts for the highest number of citations (*n* = 690), significantly more than the next highest (England with 135 citations). North America accounts for 57% of all the citations (Fig. 7).

The populations of cities ranged from 1,465 (Stevenson, WA, USA) [38] to 23,019,196 (Shanghai, China) [34]. This highlights the vast variation in population size of cities and indicates the breadth of the interpretation and definition of a city.

#### Outcome of the initiatives

Outcome was extracted and coded into; 1) no outcome stated; 2) input or process measure; 3) positive outcome measure; or 4) negative outcome. Definition for 2) input or process measure is, for example, Feyerherm et al. [39] which reported on the number of organisations that participated and carried through some subsequent action (input measure). Or, for example, Sacks and Nonas [40] who reported on the outcome of number of adults reported as eating fruit and vegetables (process measure). Definition for 3) positive outcome measure is defined as a measure of health or a measure with a direct health benefit (eg reduced smoking, reduced obesity). For example; Duncan [41] who reported a 40% reduction in emergency department visits by high users (outcome related to healthcare). Or, Coady et al. [42] who reported a 27% decrease in smokers (direct health benefit). Or Weinstein [43] that reported on reduced infant mortality. Finally, an example of a negative outcome would be De Salazar [44] who reported the initiative to reduce prevalence of cardiovascular disease in Cali (Columbia) actually increased sedentary lifestyles.

More than half (57%) did not have an outcome recorded. Thirty four percent referenced an input or process measure. Eight percent reported an outcome measure and one percent reported a negative impact as a result of the initiative.

It is not possible to describe all the initiatives in detail in this manuscript. Therefore, an excel file is provided with all records and data extraction and analysis is available in Additional file 2. This Additional file can be used as a resource for policy makers and researchers to further interrogate and build on the data.

## Discussion

There is growing interest in the actions that cities are taking to improve health and healthcare. This is in keeping with the increasing importance of cities in determining global population health. The earliest initiative was the House of Lords Select Committee *Report on Metropolitan Hospitals, Provident and Other Public Dispensaries and Charitable Institutions for the Sick Poor, *[45] published in 1892. Whilst the social context and healthcare delivery have developed significantly, the issues identified in this report, including the need for co-ordination between services and unacceptable inequalities, remain as relevant today. The collective body of evidence identified in this review, built up over the last 130 years, has hitherto been poorly documented and characterised. This may in part explain why longstanding issues remain pertinent today.

Education was the most frequently applied type of initiative (26%), with the trend increasing over time. It consistently features as one of the most reported initiatives in the last 15 years. With non-communicable diseases – often referred to as ‘life style choices’ [46, 47]—being such a high proportion (92%) of the subject areas that initiatives were seeking to address, it is perhaps not surprising that education features so strongly. Education initiatives tend to be straightforward to write up and evaluate [48], which lends them towards publication. They are also commonly associated with funding [49] that requires some kind of evaluation or report, which again lends towards publication. However, in our experience, trying to change individual behaviour without changing the system or environment within which people live does not work. The term ‘life style choices’is a misnomer. The reality is that we are strongly influenced by explicit and implicit prompts in our environment and those with the least resources have the least choice and are the most lectured.

Changes to the physical environment is linked with changing the environment within which we live. It was therefore disappointing that these types of initiatives accounted for only 21% (*n* = 336) of the total. It was also disappointing that only 21 of the 336 reported an outcome measure. These were addressing issues of obesity [50, 51] smoking [52–54] water borne infections [55–57], traffic related accidents and active travel [58–62]. These changes to physical environment were instigated by policy or legal changes in just under half (43%) of the initiatives. In considering the balance between education and system or environmental initiatives, more focus and effort should be on system and environment to ‘make the healthy choice the easy choice’.

Service reform (23% of the total citations) performed best on reporting an outcome measure (*n* = 34). These ranged from centralising heart attack and stroke services, [63, 64] injection drug user facilities for harm reduction, [65] decentralising and creating networks for care for diabetes [66] and dementia [67] to mass nicotine replacement therapy [32]. Service reform, which is defined as ‘a change or addition to a health service’, is akin to a change to the physical environment or system within which we live. It is also most directly linked to health services. Therefore, service reforms have a direct and clear link to any changes in outcomes. This is not to say the changes are easy or straightforward. Changing any provision of health service, particularly if controversial services or moving services further from a patient’s home, is likely to understandably attract public and political interest. Clinical leadership and genuine public and political engagement are key.

A relatively small number (*n* = 124) of initiatives described what was believed to be a comprehensive suite of initiatives. City health departments were most notable in leading these in a diverse range of cities including New York City (USA), [68] Geelong (Australia), [69] Barcelona (Spain), [70] Taipei (Tiawan) [71] to Nizwa (Oman) [72]. Coincidently, there were also 124 records that stated an outcome measure. It is striking that although a comprehensive approach only represented 8% of the total, they represent almost a quarter (23%) of the number with an outcome measure. There could be a number of reasons why outcome measures feature strongly when a comprehensive suite of initiatives is undertaken. A range of initiatives addressing the same subject area may be more likely to bring about a change in outcome compared to a single initiative.

City Health Departments are strongly associated with City Mayors. However, of the 1614 citations only 44 (3%) referenced a Mayor as having any kind of role. Considering Mayors are such features of cities, this was surprising. It was also notable, in general, that initiatives across all areas appeared quite piecemeal and carried out in insolation; a single initiative linked to a single health issue or risk factor without reference to a wider strategy, aim or other supporting initiatives. This could simply be a product of the way the papers were written. Indeed, there is a neat simplicity to this approach. However, the reality of the real world is that a single initiative is unlikely to making meaningful and lasting impact on long-standing issues. Cities are a meta-system with population health dictated by multiple interactions and multidirectional feedback loops. A change to one part of the system could both result in multiple unintended consequences or no impact on outcomes at all. Improving health in cities requires multiple actions, by multiple actors, at multiple levels. No single organisation, sector or initiative can solve the complex interlinked issues. The political and system leadership across a collaboration of partners by a Mayor will be crucial. The role and impact of Mayors in city residents’ health would warrant further investigation.

The initiative itself also affected how it was instigated (Table 3). It should be noted that “not stated” featured highly across all groups, ranging up to 54% in education and support and totalling 529 (33%) across all initiatives. Therefore, care should be taken with interpreting this result. The comprehensive initiatives were broadly split one fifth policy, collaboration and city health department, with 20%, 18%, 27%, respectively. Education initiatives were most associated with collaboration (15%), city health department (9%) and policy (6%). Changes made to the physical environment were strongly associated with policy (22%) and law (21%).Digital, IT and data was most commonly instigated by city health departments (21%) (Table 3).

**Table 3:** Type of initiative and how it was instigated

Type of Initiative	# of papers	%	How Intervention Instigated	How Instigated volume	How Instigated percentage	# with outcome measure	Percentage of the total of those with an outcome
Comprehensive	124	8%	Policy	25	20.2%	3	2.4%
			Collaboration	22	17.7%	1	0.8%
			Service Reform	0	0.0%	0	0.0%
			Law (4)	1	0.8%	1	0.8%
			City Health Dept	34	27.4%	17	13.7%
			Fiscal (6)	0	0.0%	0	0.0%
			Research	1	0.8%	0	0.0%
			National or philanthropc	7	5.6%	0	0.0%
			WHO Healthy Cities (9)	6	4.8%	0	0.0%
			WHO	1	0.8%	0	0.0%
			Stanford (11)	0	0.0%	0	0.0%
			Not stated	27	21.8%	7	5.6%
Education / support program	414	26%	Policy	26	6.3%	2	1.6%
			Collaboration	63	15.2%	4	3.2%
			Service Reform	9	2.2%	0	0.0%
			Law (4)	8	1.9%	0	0.0%
			City Health Dept	36	8.7%	2	1.6%
			Fiscal (6)	3	0.7%	4	3.2%
			Research	16	3.9%	0	0.0%
			National or philanthropc	19	4.6%	1	0.8%
			WHO Healthy Cities (9)	3	0.7%	0	0.0%
			WHO	2	0.5%	0	0.0%
			Stanford (11)	7	1.7%	3	2.4%
			Not stated	222	53.6%	14	11.3%
Change to physical environment	336	21%	Policy	75	22.3%	1	0.8%
			Collaboration	17	5.1%	1	0.8%
			Service Reform	4	1.2%	1	0.8%
			Law (4)	69	20.5%	4	3.2%
			City Health Dept	27	8.0%	0	0.0%
			Fiscal (6)	16	4.8%	1	0.8%
			Research	8	2.4%	0	0.0%
			National or philanthropc	10	3.0%	2	1.6%
			WHO Healthy Cities (9)	4	1.2%	0	0.0%
			WHO	1	0.3%	0	0.0%
			Stanford (11)	0	0.0%	0	0.0%
			Not stated	105	31.3%	11	8.9%
Service reform	366	23%	Policy	14	3.8%	0	0.0%
			Collaboration	47	12.8%	4	3.2%
			Service Reform	228	62.3%	25	20.2%
			Law (4)	7	1.9%	1	0.8%
			City Health Dept	31	8.5%	3	2.4%
			Fiscal (6)	18	4.9%	0	0.0%
			Research	2	0.5%	0	0.0%
			National or philanthropc	19	5.2%	1	0.8%
			WHO Healthy Cities (9)	0	0.0%	0	0.0%
			WHO	0	0.0%	0	0.0%
			Stanford (11)	0	0.0%	0	0.0%
			Not stated	0	0.0%	0	0.0%
Digital / IT / Data	92	6%	Policy	2	2.2%	0	0.0%
			Collaboration	5	5.4%	1	0.8%
			Service Reform	6	6.5%	0	0.0%
			Law (4)	0	0.0%	0	0.0%
			City Health Dept	19	20.7%	1	0.8%
			Fiscal (6)	1	1.1%	0	0.0%
			Research	7	7.6%	0	0.0%
			National or philanthropc	4	4.3%	0	0.0%
			WHO Healthy Cities (9)	3	3.3%	0	0.0%
			WHO	0	0.0%	0	0.0%
			Stanford (11)	0	0.0%	0	0.0%
			Not stated	45	48.9%	2	1.6%
Not stated / no intervention	282	17%	Policy	36	12.8%	2	1.6%
			Collaboration	27	9.6%	0	0.0%
			Service Reform	0	0.0%	2	1.6%
			Law (4)	6	2.1%		0.0%
			City Health Dept	16	5.7%	0	0.0%
			Fiscal (6)	0	0.0%	0	0.0%
			Research	21	7.4%	0	0.0%
			National or philanthropc	9	3.2%	0	0.0%
			WHO Healthy Cities (9)	35	12.4%	0	0.0%
			WHO	2	0.7%	0	0.0%
			Stanford (11)	0	0.0%	0	0.0%
			Not stated	130	46.1%	2	1.6%

Total citations with an outcome measure: 124

The majority of papers were from North America (57% of total, almost double the total of Europe) and in particular New York City which alone accounted for 17% (*n* = 279) of the total, more than the entire WHO Healthy City output found. It is not clear if this is because New York City is taking significant steps to improve health and healthcare or they are publishing a lot of their work, or both. This features in the bias and limitations identified in the section below. It also highlights the opportunity for further study through a survey of city health departments to get a more complete picture of the initiatives that cities are undertaking to improve population health.

The breadth of subject areas being addressed from a number of different perspectives and the interrelated nature of the subject areas is striking. For example, diet is known to impact on obesity, [73] and cancer [74], and cardiovascular [75] disease. Obesity is known to impact on cancer, [76] cardiovascular, [77] mental health [78] and mental health is known to impact on obesity [79]. Therefore, an initiative to improve diet could be an initiative to address obesity, cancer, cardiovascular disease, mental health or health generally.

The complexity of a range of subject areas being addressed could be simplified when the major causes of chronic diseases are well known. Indeed, the four main risk factors (tobacco use; harmful alcohol use; physical inactivity and unhealthy diet) account for at least 80% of all heart disease, stroke and type 2 diabetes and 40% of cancers [80]. The authors would add a fifth risk factor; *planetary health.* Planetary health is widely acknowledged to be a significant risk to human health [81–83] particularly impacting cities [84, 85]. The health risk factors are also directly linked to planetary health factors, for example diet [86] and evidence shows polices to promote sustainable food supply can improve personal and planetary health [87]. The authors would call these ‘The Vital 5’. They are the 5 most important health risk factors. These ‘Vital 5’ are most concentrated in deprived areas [88] and show the greatest increase is in low and middle income countries, even faster than has historically occurred in high income countries [89]. Therefore, action on these ‘Vital 5’ will be action on inequalities. Every city should develop a comprehensive strategy and action plan to address these ‘Vital 5’ of tobacco use; harmful alcohol use; physical inactivity, unhealthy diet and planetary health.

The 20th century was defined by breakthroughs in communicable diseases. Communicable diseases are certainly not eradicated; HIV [90–92] and TB [93–95] can still be seen to be particular issues in cities. The Covid-19 pandemic has also disproportionately impacted cities [12]. However, Covid-19 has also reminded us of the importance of these Vital 5. It is known that smoking, [96] alcohol, [97] poor diet, [98] physical inactivity [99] and air quality [100], all contribute to worse outcomes of Covid-19. They also contribute to other factors which have worse outcomes of Covid-19, such as high blood pressure [101] and obesity [102]. Crucially, it is also know that damaging the environment will make future pandemics more likely [103, 104]. If cities were to focus on these Vital 5 they would be improving immediate and long term population health.

This study has several limitations. The deliberate decision was taken to be as broad and inclusive as possible in the papers included. Mays et al. [105] and Strech and Tilburt [106] identify the tension between using only academically rigorous, but therefore less, research and the inclusion of other sources of data, which increase the volume, but that are more susceptible to bias and therefore risk contaminating results. Because of the volume of papers and restrictions on time only, the title and abstract were used in the data extraction. Formal quality assessment of the studies was not undertaken. Only studies in English language were included. A large volume of citations were from one city (New York City) which could introduce bias. Finally, given the heterogeneity of the study designs and data sources, meta-analysis was not undertaken. The authors acknowledge that despite a comprehensive and systematic approach they may have missed relevant documentation.

The authors believe non-communicable diseases will be *the* issue of the 21st century, accounting for over 40 million annual deaths globally (72% of total deaths) [107]. The Vital 5; tobacco use; harmful alcohol use; physical inactivity, unhealthy diet and planetary health impact on both non-communicable diseases and the likelihood of future communicable pandemics. The progress made in the treatment and prevention of HIV from death sentence 20 years ago to entirely treatable and preventable long-term condition, plus the rapid response to Covid-19 demonstrates what could be achieved to eliminate these ‘Vital 5’ with collective determination and resources. Cities have demonstrated they are well placed to take this action.

## Conclusions

This global systematic scoping review provides an overview of the existing evidence of city initiatives to improve health and healthcare. Based on the findings, a summary of key findings and recommended policy actions is provided below. This, with the excel file with the 1614 records and associated extracted data (Additional file 2), will be a useful resource for academics and policy makers interested in improving urban health. The outputs of this systematic scoping review can be used to inform urban health policy for the next decade and shape future research. The role of Mayors and the real world understanding of the initiatives that city health departments are undertaking to improve population health warrants particular attention.

Key findings and recommended policy actions:

- **Key Finding:** There is a large range of health issues and risk factors being addressed from a large number of perspectives. The overwhelming majority of the issues and initiative are addressing ‘non-communicable disease’ risk factors that are interrelated and overlapping.
- **Recommended Policy Action:** The same level of ambition that was applied to HIV and Covid-19 should be applied to the ‘Vital 5’ risk factors; smoking, alcohol, physical inactivity, unhealthy diet and planetary health.
- **Key Finding:** Education was the most commonly reported initiative.
- **Recommended Policy Action:** The term ‘lifestyle choices’ is a misnomer. More focus and resource should be put on system and environmental changes to ‘make the healthy choice the easiest choice’
- **Key Finding:** City health departments are making an increasing contribution in improving city population health.
- **Recommended Policy Action:** Cities should collaborate and share learning in real world experience of initiatives to improve urban health.
- **Key Finding:** The contribution of Mayors in improving health and healthcare appears to be limited
- **Recommended Policy Action:** The role and impact of Mayors in city populations would warrant further study
- **Key Finding:** New York City stands out with nearly one fifth (18%) of the total citations.
- **Recommended Policy Action:** Further real world research to understand and get a comprehensive view on what cities are doing to improve population health would be of value.

## Supplementary Information

**Additional file 1.** Database Search Strings.

**Additional file 2.**

## Data Availability

The data generated from this review is provided in the supplementary information files.

## Abbreviations

Covid-19: Coronavirus disease 2019
PRISMA: Preferred Reporting Items for Systematic Reviews and Meta-Analyses
PROSPERO: International prospective register of systematic reviews
HLP: Healthy London Partnership.
HIV: Human immunodeficiency virus
TB: Tuberculosis
WHO: World Health Organisation
IT: Information Technology

## Footnotes

1 See acknowledgements for full list.
