# Supplementary material for: Healthy city: global systematic scoping review of city initiatives to improve health with policy recommendations
Source: BMC Public Health. 2023 Jul 1;23:1277. doi: 10.1186/s12889-023-15908-0 (PMC10314468; doi:10.1186/s12889-023-15908-0)
Supplement: Supplementary file 1 — Additional file 1. Database Search Strings. [file 12889_2023_15908_MOESM1_ESM.docx]

Appendix A – Database Search Strings

**Embase classic + embase 1947**

1. city/

2. city planning/

3. (city or cities).ti,ab.

4. 1 or 2 or 3

5. "transform*".ti,ab.

6. (improve* or improving).ti,ab.

7. "reform*".ti,ab.

8. revolution.ti,ab.

9. "moderni*".ti,ab.

10. (change* or changing).ti,ab.

11. 5 or 6 or 7 or 8 or 9 or 10

12. health/ or adolescent health/ or child health/ or dental health/ or family health/ or health status/ or men's health/ or mental health/ or minority health/ or nutritional health/ or reproductive health/ or sexual health/ or veterans health/ or women's health/

13. public health/

14. "health*".ti,ab.

15. 12 or 13 or 14

16. 4 and 11 and 15

**Ovid MEDLINE(R) In-Process & Other Non-Indexed Citations and Ovid MEDLINE(R)** 1946 to Present

1. exp Cities/

2. (city or cities).ab,ti.

3. 1 and 2

4. exp Health Care Reform/

5. "transform*".ab,ti.

6. (improve* or improving).ab,ti.

7. (change* or changing).ab,ti.

8. "reform*".ab,ti.

9. "revolution*".ab,ti.

10. "moderni*".ab,ti.

11. 4 or 5 or 6 or 7 or 8 or 9 or 10

12. exp Health Services/

13. exp Public Health/

14. "health*".ab,ti.

15. 12 or 13 or 14

16. 3 and 11 and 15

**Central (Cochrane Central Register for controlled trials**

[http://onlinelibrary.wiley.com/cochranelibrary/](http://onlinelibrary.wiley.com/cochranelibrary/search/advanced?hiddenFields.strategySortBy=last-modified-date;desc&hiddenFields.showStrategies=false&hiddenFields.containerId=1306905460302935290&hiddenFields.etag=9148908297592433051&meshOrBasicAppended=true)

ID Search Hits

#1 city or cities:ti,ab,kw

#2 transform* or improve* or improving or change* or changing or reform* or revolution or moderni*

#3 health*

#4 #1 and #2 and #3

**Scopus**

Searched title, abstract, keyword on: City or cities AND transform* or improve* or improving or change* or changing or reform* or revolution or moderni* AND health*

Limited to English language

Excluded the following subject areas:

Biochemistry, genetics and molecular biology and engineering and agricultural and biological sciences and earth and planetary sciences and computer sciences and physics and astronomy and chemical engineering and chemistry and veterinary, energy, arts and humanities, chemistry, mathematics, decision sciences, material sciences, chemical enginerring.

The search script was:

( TITLE-ABS-KEY ( **city**  OR  **cities** )  AND  TITLE-ABS-KEY ( **transform***  OR  **improve***  OR  **improving**  OR  **change***  OR  **changing**  OR  **reform***  OR  **revolution**  OR  **moderni*** )  AND  TITLE-ABS-KEY ( **health*** ) )  AND  ( LIMIT-TO ( LANGUAGE ,  **"English"** ) )  AND  ( EXCLUDE ( SUBJAREA ,  **"BIOC"** )  OR  EXCLUDE ( SUBJAREA ,  **"ENGI"** )  OR  EXCLUDE ( SUBJAREA ,  **"AGRI"** )  OR  EXCLUDE ( SUBJAREA ,  **"EART"** )  OR  EXCLUDE ( SUBJAREA ,  **"COMP"** )  OR  EXCLUDE ( SUBJAREA ,  **"PHYS"** )  OR  EXCLUDE ( SUBJAREA ,  **"CENG"** )  OR  EXCLUDE ( SUBJAREA ,  **"CHEM"** )  OR  EXCLUDE ( SUBJAREA ,  **"VETE"** ) )

**Campbell Library**

Direct through their website: <http://www.campbellcollaboration.org/lib/>

searched ‘keywords’ on: City or cities AND transform* or improve* or improving or change* or changing or reform* or revolution or moderni* AND health*

this produced no hits

**CINAHL**

Searched through link from NHS Evidence and logged in through Open Athens. This covers 1981 – present.

| 3 | (city OR cities).ti,ab |  |
| --- | --- | --- |
| 4 | (transform* OR improve* OR improving OR change* OR changing OR reform* OR revolution OR moderni*).ti,ab |  |
| 5 | health*.ti,ab |  |
| 6 | 3 AND 4 AND 5 |  |

**Health Business Elite**

Searched through link from NHS Evidence and logged in through Open Athens. This covers 1922 – present.

Search query:

1 (city OR cities).ti,ab

2 HEALTH BUSINESS ELITE (transform* OR improve* OR improving OR change* OR changing OR reform* OR revolution OR moderni*).ti,ab

3 HEALTH BUSINESS ELITE health*.ti,ab

4 HEALTH BUSINESS ELITE 1 AND 2 AND 3

This produced 1341 hits. These were transferred to Endnote and filed on the 11^th^ Feb 2016

Set up weekly email alert too.

**Health Management Information Consortium (HMIC)**

Searched through link from NHS Evidence and logged in through Open Athens. This covers 1979 – present

Search term:

1 (city OR cities).ti,ab

2(transform* OR improve* OR improving OR change* OR changing OR reform* OR revolution OR moderni*).ti,ab

3 health*.ti,ab

4 1 AND 2 AND 3

**PyschINFO**

Run through OViD interface.

1. urban environments/

2. (city or cities).ab,ti.

3. 1 or 2

4. exp Health Care Reform/

5. "transform*".ab,ti.

6. (improve* or improving).ab,ti.

7. "reform*".ab,ti.

8. revolution.ab,ti.

9. "moderni*".ab,ti.

10. (change* or changing).ab,ti.

11. 4 or 5 or 6 or 7 or 8 or 9 or 10

12. exp health/

13. "health*".ab,ti.

14. 12 or 13

15. 3 and 11 and 14

**Centre for Reviews and Dissemination (PROSPERO)**

Searched directly through: [www.crd.york.ac.uk/PROSPERO](http://www.crd.york.ac.uk/PROSPERO). Search term:

1 (city OR cities).ti,ab

2(transform* OR improve* OR improving OR change* OR changing OR reform* OR revolution OR moderni*).ti,ab

3 health*.ti,ab

4 1 AND 2 AND 3

Limited to title. This produced 0 hits.

Change search field to ‘all fields’ and still produced 0 hits.
